# A mTurquoise-Based cAMP Sensor for Both FLIM and Ratiometric Read-Out Has Improved Dynamic Range

**DOI:** 10.1371/journal.pone.0019170

**Published:** 2011-04-29

**Authors:** Jeffrey B. Klarenbeek, Joachim Goedhart, Mark A. Hink, Theodorus W. J. Gadella, Kees Jalink

**Affiliations:** 1 Division of Cell Biology, The Netherlands Cancer Institute, Amsterdam, The Netherlands; 2 Section of Molecular Cytology, Swammerdam Institute for Life Sciences, University of Amsterdam, Amsterdam, The Netherlands; 3 van Leeuwenhoek Centre of Advanced Microscopy, Amsterdam, The Netherlands; University of Birmingham, United Kingdom

## Abstract

FRET-based sensors for cyclic Adenosine Mono Phosphate (cAMP) have revolutionized the way in which this important intracellular messenger is studied. The currently prevailing sensors consist of the cAMP-binding protein Epac1, sandwiched between suitable donor- and acceptor fluorescent proteins (FPs). Through a conformational change in Epac1, alterations in cellular cAMP levels lead to a change in FRET that is most commonly detected by either Fluorescence Lifetime Imaging (FLIM) or by Sensitized Emission (SE), e.g., by simple ratio-imaging. We recently reported a range of different Epac-based cAMP sensors with high dynamic range and signal-to-noise ratio. We showed that constructs with cyan FP as donor are optimal for readout by SE, whereas other constructs with green FP donors appeared much more suited for FLIM detection. In this study, we present a new cAMP sensor, termed ^T^Epac^VV^, which employs mTurquoise as donor. Spectrally very similar to CFP, mTurquoise has about doubled quantum efficiency and unlike CFP, its fluorescence decay is strictly single-exponential. We show that ^T^Epac^VV^ appears optimal for detection both by FLIM and SE, that it has outstanding FRET span and signal-to-noise ratio, and improved photostability. Hence, ^T^Epac^VV^ should become the cAMP sensor of choice for new experiments, both for FLIM and ratiometric detection.

## Introduction

Fluorescent biosensors have become important tools to study signaling events with high spatial and temporal resolution in living cells. Biosensors are genetically engineered constructs that report on protein conformation or activation status by alterations in spectral properties. FRET (Fluorescence- or Förster- Resonance Energy Transfer) is particularly popular as readout in such sensors. FRET is the non-radiative energy transfer from a suitable donor fluorophore to an acceptor. It occurs exclusively when donor and acceptor are in close proximity (<10 nm) and are properly oriented with respect to each other. FRET can be conveniently recorded by several techniques[1]. For time-lapses in living cells, the most common techniques are Fluorescence Lifetime IMaging (FLIM) and recording of Sensitized Emission (SE), each of which have their own strengths and weaknesses. FLIM, which records the fluorescence decay of the donor, reveals FRET as it shortens the decay time constant, τ. FLIM is precise and straight-forward, but as lifetimes of fluorescent proteins are in the order of a few ns, it requires dedicated and complex equipment. Alternatively, FRET can be read out by detecting sensitized emission, i.e. the fluorescence stemming from acceptors that are excited by resonance. SE can be detected by ratiometry on simple, widely available equipment but is usually not quantitative, unless rather involved corrections are carried out. This disadvantage can be largely overcome if end-point calibrations are possible.

We and others recently reported Epac-based FRET sensors for the second messenger cyclic adenosine monophosphate (cAMP) [2]–[4]. Our version, termed CFP-Epac(CD, ΔDEP)-YFP, consist of part of the cAMP-binding protein Epac1[5], which we stripped of the membrane-targeting DEP-sequence (ΔDEP) and made catalytically dead (CD), and then sandwiched between the donor Cyan Fluorescent Protein (CFP) and acceptor Yellow Fluorescent Protein (YFP). On cAMP binding, Epac undergoes a large conformational change which causes a robust decrease in FRET. As this FRET sensor is cytoplasmic, responds to [cAMP] changes in the physiological range (∼0.1–100 µM) and its expression is well tolerated by cells, it has rapidly become the sensor of choice for live-cell experiments. In a follow-up study we optimized the Epac sensor by systematically exchanging CFP and YFP for many of the spectrally variant fluorescent proteins available [6]. This study identified not one, but rather two improved ‘second generation’ versions. mECFPΔ-Epac(CD, ΔDEP)-^cp173^Venus-Venus, a Cyan-Yellow variant, displayed by far the best FRET span and signal-to-noise ratio, but due to the complex decay kinetics [7], [8] and moderate quantum yield of mECFP it is less suitable for quantitative FLIM. We therefore also generated a version with the bright mono-exponentially decaying donor Green Fluorescent Protein (GFP). After optimization, GFPΔ-Epac(CD, ΔDEP)-mRFP proved superior for FLIM detections. Although these sensors each are state-of-the-art in performance [6], identification of a single version suited for both FLIM and SE detection would give the researcher free choice of detection method as well as minimize necessary efforts of subcloning and characterization of cell lines.

Here we report cloning and characterization of a ‘third generation’ cAMP sensor, mTurquoiseΔ-Epac(CD, ΔDEP)-^cp173^Venus-Venus, that performs optimal in both FLIM and SE detection schemes. It is generated by replacing mECFP with mTurquoise, a novel, very bright and single-exponentially decaying CFP variant that we recently identified [9]. The more-than-doubled quantum yield of mTurquoise yields exceptional signal-to-noise and allows minimizing excitation damage. We also demonstrate that the new sensor (called ^T^Epac^VV^) is well suited for both time-domain and frequency-domain FLIM detection, showing a robust change of up to 30% in FRET efficiency in individual cells. Finally, in ratiometric SE assays the dynamic range of the new sensor also significantly outperforms previous versions. In conclusion, ^T^Epac^VV^ is the cAMP sensor of choice for new experiments, both for FLIM and ratiometric detection.

## Results and Discussion

### Generation of constructs and expression in cells

**Figure 1:**
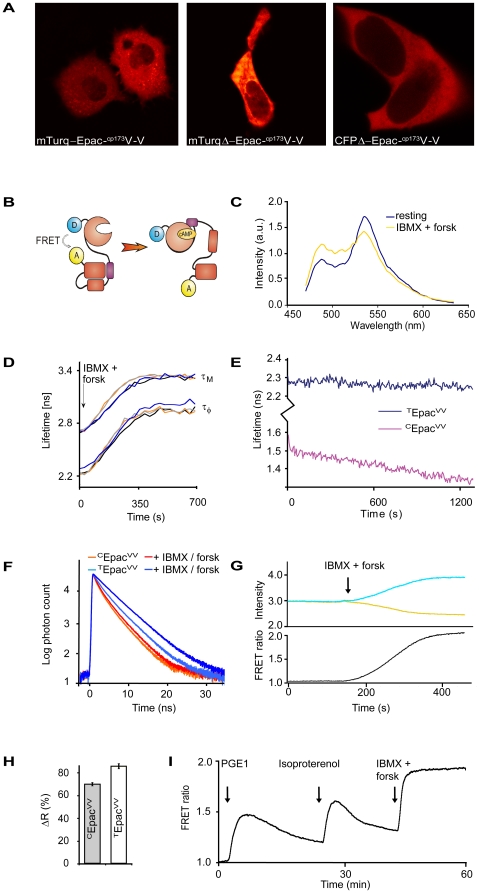
Characterization of ^T^Epac^VV^ as a FRET sensor for cAMP. (A) Hek293 cells expressing indicated constructs show cytosolic localization. Confocal images were taken 18 hours after transfection. (B) cAMP-induced conformational switch in ^T^Epac^VV^ causes drop in FRET. (C) Live-cell emission spectra of ^T^Epac^VV^ in rest and after stimulation with IBMX and forskolin (forsk) to saturate cytosolic cAMP levels. Spectra (average of 6 individual cells) are normalized with respect to the isosbestic point at 505 nm. (D) Performance of ^T^Epac^VV^ in live-cell FLIM detections. Shown is a representative time-lapse recording of τ_φ_ and τ_m_ displaying a large change in FRET upon addition of IBMX and forskolin. Changes are shown for four individual cells present in the same microscope image. (E) Time-lapse FLIM recordings from cells showing much increased stability of ^T^Epac^VV^ (dark blue) as compared to ^C^Epac^VV^ (violet trace). Each trace depicts 200 FLIM recordings consisting of 12 phase images for a total of 2400 images. Exposure time was 200 ms at ∼0.8 mW output power at the image plane. Traces are average of n = 6 single-cell determinations. (F) Log/linear plot of TCSPC data illustrates close-to-single-exponential decay of fluorescence in ^T^Epac^VV^ in rest (dark blue trace). Saturation of cAMP levels with IBMX + forskolin (bright blue trace) causes the decay time constant to increase from 2.53±0.02 to 2.97±0.03 ns in this experiment. The red trace shows distinctly bi-exponential decay of ^C^Epac^VV^ for comparison. Representative example of n>12 experiments. (G) Analysis of cAMP-induced FRET changes in ^T^Epac^VV^ analyzed by ratiometry. Changes in emission intensity in the CFP channel (blue) and YFP channel (yellow) demonstrate the exceptional S/N and FRET span of ^T^Epac^VV^. The black trace depicts the ratio CFP/YFP. Sampling interval was 0.5 s. (H) Quantification of ratiometric changes, showing the increased ratio span of our novel sensor. Data are mean ± s.e.m. from at least 12 independent determinations. (I) Typical FRET trace, obtained from a single Hek293 cell expressing ^T^Epac^VV^. Following recording of a baseline, the preparation was stimulated with prostaglandin E1 (PGE1, 5 µM) and isoproterenol (10 µM) as indicated. Calibration was with IBMX + forskolin. Traces of this quality were routinely obtained. Note that all experiments were carried out at 37°C except for the frequency-domain FLIM experiments, which were done at room temperature. This temperature difference explains the approximately 50% slower risetimes of agonist-induced cAMP changes that were generally observed in frequency-domain FLIM measurements. Conversely, ratiometric detections carried out at room temperature were also slow.

In our previous study [6], we showed that a double acceptor consisting of a tandem of Venus and a circular permutation of Venus (^cp173^Venus; [10]) proved optimal for FRET with mECFP. We generated a new Epac-based cAMP sensor by combining our bright and single exponential donor variant mTurquoise [9] with this acceptor, yielding mTurquoise-Epac(CD, ΔDEP)-^cp173^Venus-Venus (see M&M). Since we also showed that deletion of 11 C-terminal amino-acids from mECFP further boosted FRET, a similar truncation was applied to mTurquoise, yielding mTurquoiseΔ-Epac(CD, ΔDEP)-^cp173^Venus-Venus. Constructs were transfected in Hek293 cells for characterization. Both constructs display a mainly cytosolic expression with some fluorescence discernible in the nucleus (Fig. 1A, left and middle panels), similar to the parental construct (right panel). Expression, even to very high levels, was well-tolerated by the cells. We therefore set out to further characterize the constructs both for spectral properties and for use as cAMP sensors.

### Emission spectra

The emission spectra of mTurquoiseΔ-Epac(CD, ΔDEP)-^cp173^Venus-Venus (see Fig. 1B, C) and mTurquoise-Epac(CD, ΔDEP)-^cp173^Venus-Venus appeared identical. However, in good agreement with the much higher brightness of mTurquoise [9], these spectra showed a more prominent peak at ∼480 nm in comparison to mECFPΔ-Epac(CD, ΔDEP)-^cp173^Venus-Venus (compare Fig. 1C with Fig. 4B in van der Krogt et al.). Addition of the adenylyl cyclase activator forskolin (25 µM) and the phosphodiesterase inhibitor IBMX (100 µM), which together cause maximal and sustained cAMP elevation in these cells, resulted in a sizeable decrease in FRET, as judged from the increase in mTurquoise emission and concomitant decrease in YFP emission (Fig. 1C). Average IBMX + forskolin-induced changes in the ratio of mTurquoise to YFP peaks (ΔR), measured at 478 and 529 nm, respectively, were 82.2±1.4% for mTurquoiseΔ-Epac(CD, ΔDEP)-^cp173^Venus-Venus, and 40.0±1.7% (n = 22) for mTurquoise-EPAC(CD, ΔDEP)-^cp173^Venus-Venus. Since the truncated Turquoise mutant outperformed the version with full-length mTurquoise donor throughout our studies, we further focus in this report on mTurquoiseΔ-Epac(CD, ΔDEP)-^cp173^Venus-Venus, which will be termed ^T^Epac^VV^ henceforth.

### Fluorescence lifetime recordings

As a prime goal of this study was to generate a single sensor optimal for both spectral and FLIM detection, we next tested performance of ^T^Epac^VV^ in lifetime measurements. Hek293 cells expressing either ^T^Epac^VV^ or the parental construct mECFPΔ-Epac(CD, ΔDEP)-^cp173^Venus-Venus (hereafter called ^C^Epac^VV^) were tested by frequency-domain FLIM measurements [11]. Consistent with the high FRET levels of Epac in closed conformation [12], the lifetime of ^T^Epac^VV^ in resting state was significantly lower than that of free mTurquoise (τ_φ_ 2.28 ns versus 3.71 ns; compare Table 1). Addition of IBMX and forskolin caused an increase to 3.02 ns (Table 1; Fig. 1D and Movie S1). Such a striking change in lifetime is usually only observed in protease biosensors in which donor and acceptor are physically separated upon cleavage [13]. Thus, cAMP induced a robust drop in FRET, averaging about 0.74±0.08 ns in these experiments. In contrast, cAMP-induced lifetime changes in ^C^Epac^VV^ amounted to just 0.38±0.10 ns. We also noted that use of ^T^Epac^VV^ in time-lapse FLIM measurements yielded much more stable FRET readings than use of ^C^Epac^VV^ (Fig. 1E). For these experiments, 200 FRET determinations of 12 phase images each were recorded in time-lapse mode from unstimulated cells expressing either sensor. In ^C^Epac^VV^ this protocol typically causes a moderate exposure-dependent shift in apparent FRET values, which is probably due to photochromic effects. This ‘FLIM run-off’ is also seen in many other FRET sensors (KJ & JK, unpublished observations) as well as in free CFP and it likely underlies some of the variability encountered in FRET determinations. Importantly, ^T^Epac^VV^ proved almost completely insensitive to FLIM run-off.

**Table 1:** FRET efficiency for Epac sensors determined by frequency-domain FLIM.

Donor	Sensor ID	IBMX + forsk.	n^1^	τ_φ_ [ns]^2^	τ_M_ [ns]^3^	E τ_φ_ [%]^4^	E τ_M_ [%]^4^
mECFPΔ	H96	**−**	16	1.64±0.04	2.18±0.06	29	25
		**+**	18	2.02±0.11	2.61±0.11	12	13
mTurqΔ	H74	**−**	22	2.28±0.04	2.79±0.09	38	27
		**+**	21	3.02±0.08	3.40±0.07	18	10

Measured fluorescence lifetimes and calculated FRET efficiencies for the different Epac-constructs before (−) and after (+) stimulation with IBMX and Forskolin for 10 min.

1 n, number of cells;

2 τ_φ_ average phase lifetime ± s.d.;

3 τ_M_, average modulation lifetime ± s.d.;

4 E, average FRET efficiency calculated from τ_φ_ or τ_M_ according to (1- τ_DA_/τ_D_) * 100%, using τ_D_ values of 3.71 ns and 3.80 ns for mTurquoise and τ_D_ values of 2.28 ns and 2.99 ns for mECFP, for phase and modulation lifetimes respectively [9].

To directly assess the decay kinetics of these constructs, we employed Time Correlated Single Photon counting (TCSPC; Fig. 1F). As expected, ^C^Epac^VV^ proved distinctly multi-exponential, both in resting cells and upon elevation of cAMP levels. In contrast, decay of cAMP-saturated ^T^Epac^VV^ was well-fit with a single exponent (χ^2^ between 1.0 and 1.6), reflecting a single species of unfolded Epac in the population. At intermediate levels of cAMP, a fast and a slow decay component (representing cAMP-bound, unfolded sensors, and free, folded sensors) were clearly distinguishable (data not shown). Analysis revealed that in unstimulated cells, a small percentage of sensors are bound to cAMP, as witnessed from the non-zero amplitude of the fast component. In support of this interpretation, lowering cAMP levels to below resting state by stimulation of Gα_i_-coupled LPA receptors further diminished the fast component and significantly increased the fit with a single exponential (χ^2^_resting_ 3.5 and χ^2^_LPA_ 1.8). Lifetimes as determined by TCSPC were generally in reasonable agreement with the frequency-domain data and confirmed the robust change in FRET following elevation of cAMP levels (Fig. 1F). We conclude that ^T^Epac^VV^ performs well for both static FRET-FLIM detections and for time-lapse FLIM recording.

### Ratiometry

FRET sensors for cAMP are most commonly read out by ratiometric time-lapse recording. We therefore compared performance of ^T^Epac^VV^ and ^C^Epac^VV^ by simultaneously detecting CFP and YFP emission on a dual-photometer system (see M&M). Following recording of a baseline, IBMX and forskolin were added and changes in ratio were observed until a stable plateau was reached (Fig. 1G). Not surprisingly, dynamics of FRET changes were identical for both sensors, indicating that the mTurquoise substitution did not affect the cAMP-sensing moiety (not shown). Remarkably, however, ^T^Epac^VV^ significantly outperformed the already robust response of ^C^Epac^VV^ (Fig. 1H). Moreover, the approximately doubled brightness of ^T^Epac^VV^ allows routine recording of traces with exceptional S/N ratio (Fig. 1G). Alternatively, the increased brightness may be traded against approximately two-fold lower excitation intensity to minimize photodamage in long timelapse experiments. In conclusion, among all our sensors ^T^Epac^VV^ is optimal for ratiometry too.

### Performance in biological applications

Finally, we tested performance of ^T^Epac^VV^ in a series of long time-lapse experiments with more physiological stimuli. In HEK293 cells, administration of prostaglandin E1 (PGE1; 5 µM) caused a large change in FRET; see Fig. 1I for an example. Subsequent addition of isoproterenol (10 µM), which activates the β-adrenergic receptor to stimulate adenylate cyclase via the G protein G_s_ [14] evoked a second transient in these cells. Finally, IBMX and forskolin were added to calibrate the response. Similar responses were observed in other cell lines tested, including N1E-115 mouse neuroblastoma cells and HeLa human cervical cancer cells. These experiments show that ^T^Epac^VV^ performs well in physiological assays, underlining its status as ruling champion cAMP sensor.

### Concluding remarks

We here report cloning and characterization of ^T^Epac^VV^, an improved successor to our line of Epac-based cAMP sensors [15], [6]. Advantages of the new sensor are that (i) a single construct performs optimally for both readout by FLIM and by sensitized emission (ratiometry), thus reducing the work load for e.g. generation of cell lines and transgenic animals; (ii) it has an increased FRET span both for FLIM and for ratiometric detections; (iii) it is about twice as bright as the parental construct; and (iv) the Venus acceptors are devoid of the maturation problems that are commonly observed in the red FP acceptors present in green-to-red FRET sensors.

When compared to ^C^Epac^VV^, ^T^Epac^VV^ exhibits about 35% increased change in lifetime as detected by frequency-domain FLIM measurements and TCSPC. It is interesting to note that this difference is larger than the improvement in ratio change (Fig. 1H shows ∼22% improved ratio change). This is not unexpected because the improved brightness of mTurquoise also causes an increase in leakthrough of donor signal into the acceptor channel. cAMP-induced decreases in FRET thus cause opposing signals in the acceptor channel, i.e. an increase in donor leakthrough that partly counteracts the decrease in sensitized emission.

Another favorable characteristic of ^T^Epac^VV^ is the virtual absence of FLIM run-off (Fig. 1E). In practical FRET measurements, this resulted in much less cell-to-cell variability and it allowed collection of meaningful FLIM data in prolonged time-lapses. Two factors are likely to contribute to this effect. First, mTurquoise is superior to CFP in that it shows considerably less photobleaching [9]. Second, the higher quantum yield of mTurquoise ensures that a more prominent fraction of the absorbed energy is emitted as fluorescence or transferred by FRET to acceptors. This indicates that less energy is lost to internal conversion processes (heat dissipation) that may cause photochromism. Irrespective of the precise contribution of these factors, they add to make ^T^Epac^VV^ exceptionally suited for FLIM determinations.

## Materials and Methods

### Cell culture and transfection

Hek293 embryonal kidney cells (American Type Culture Collection crl-1573), N1E-115 mouse neuroblastoma cells (crl-2263) and human HeLa servical cancer cells (ccl-2) were cultured in DMEM supplemented with 10% FCS and antibiotics. Cells were seeded on 25-mm coverslips in six-well plates and transfected with 1 µg DNA per well using calcium phosphate or fugene transfection agent.

### Constructs and materials

From the starting material CFPΔ-EPAC-^cp173^Venus-Venus [6] and mTurquoise we generated mTurquoise-EPAC-^cp173^Venus-Venus by inserting mTurquoise with forward PCR primer GATCGGCGGCCGCAATGGTGAGCAAGGGCGAGGAG and reverse primer GATCGATATCCCTTGTACAGCTCGTCCATGCC. The truncation mutant lacking the last eleven amino acids (GITGMDELYK), mTurquoiseΔ was generated by PCR using reverse primer AAAGGATATCGGGCGGCGGTCACGAACT. PCR products and plasmids containing the CFPΔ were cut with NotI and EcoRV. All constructs were checked by sequence analysis.

IBMX and forskolin were obtained from Calbiochem-Novabiochem Corp. (La Jolla, CA). PGE1 and isoproterenol were obtained from Sigma-Aldrich (Zwijndrecht, The Netherlands).

### Confocal microscopy

Experiments were performed in HEPES buffered saline (containing 140 mM NaCl, 5 mM KCl, 1 mM MgCl_2_, 1 mM CaCl_2_, 10 mM glucose, 10 mM HEPES) pH = 7.2 at 37°C. Images were taken using a Leica TCS-SP5 confocal point-scanning microscope (Mannheim; Germany) using a 63x, 1.3 N.A. glycerol immersion objective. Donor excitation was with the 442 nm HeCd laser; donor emission was collected between 450 and 505 nm and acceptor emission between 510 and 600 nm by setting the SP5 spectrometer accordingly. The spectral emission scans presented in Fig. 1C were taken in xyλ mode, scanning between 460 nm and 630 nm with a 5-nm step size. Agonists and inhibitors were added from concentrated stocks.

### Ratiometric FRET analysis

Cells on coverslips were placed on a thermostatted (37°C) inverted Nikon Diaphot microscope and excited at 425 nm. Donor and acceptor emission was detected simultaneously with two photomultipliers, using a 505 nm beamsplitter and optical filters: 470±20 nm (CFP) and 530±25 nm (YFP). Signals were digitized and FRET was expressed as the ratio between donor and acceptor signals. The FRET value was set at 1 at the onset of the experiment. Cells were stimulated with 25 µM forskolin and 100 µM IBMX to maximally raise the cAMP levels. Data from 6–15 cells per experiment are presented as mean ± s.e.m.

### Fluorescence Lifetime Imaging

Frequency domain FLIM measurements were essentially performed as described [9] using the wide-field frequency domain approach with a 440 nm modulated diode laser (LDH-M-C-440, PicoQuant) and a RF-modulated image intensifier (Lambert Instruments II18MD) coupled to a CCD camera (Photometrics HQ) as detector. The modulation frequency was set to 75 MHz and a 40x objective (Plan Apochromat NA 1.4 oil) was used. The light was reflected by a 455DCLP dichroic mirror and the CFP emission was passed through a D480/40 band-pass emission filter (Chroma Technology). Eighteen phase images with an exposure time of 50–100 ms seconds were acquired. From the phase sequence an intensity (DC) image and the phase and modulation lifetime image are calculated using Matlab macros. From these data, the average lifetime of individual cells is determined using ImageJ (http://rsb.info.nih.gov/ij/). Subsequently, average phase and modulation lifetimes are calculated. For the presentation of lifetime maps, a 5×5 smooth filter is applied to the raw data. The false-color lifetime maps and 1D and 2D histograms are generated by an ImageJ macro. These experiments were carried out at room temperature.

Time-domain FLIM experiments were performed by TCSPC on the Leica SP5 system using Becker and Hickl (Berlin) TCSPC hard- and software and a Picoquant (Berlin) 405 nm pulsed diode laser. Laser intensity was set to the minimal value that just produced reliable pulses and repetition rate was 40 MHz. FRET efficiency E was calculated as E  = 1- τ_DA_/τ_D_. All data were fitted both with one and two exponents to check for optimal fits, excluding the pulse (first 1.1 ns) from the fit region. TCSPC experiments were at 37°C.

## Supporting Information

Movie S1 **Increase in fluorescence lifetime of ^T^Epac^VV^ upon cAMP-induced sensor unfolding.** The fluorescence lifetime of the mTurquoise donor in ^T^Epac^VV^ is strongly increased when intracellular cAMP levels are raised with IBMX/forskolin. The analysis of the FLIM time-lapse experiment shows (upper row from left to right), mTurquoise intensity, phase lifetime map, phase lifetime map overlaid with the intensity, a histogram depicting the phase lifetime distribution, and (lower row from left to right) modulation lifetime map, modulation lifetime map overlaid with the intensity, a histogram depicting the modulation lifetime distribution. The lifetime maps are color-coded according to the LUT shown in the histogram and the lifetime/intensity overlays according to that shown in the lower left corner. Quantification of the fluorescence lifetime, as measured by frequency domain FLIM, over time is shown in figure 1D of the manuscript.
(AVI)
